# Primary Tracheal Lymphoma Mimicking Asthma Exacerbation

**DOI:** 10.1002/rcr2.70810

**Published:** 2026-11-02

**Authors:** Keigo Uchimura, Sumitaka Kitajima, Makoto Hirai, Teruaki Ishida

**Affiliations:** ^1^ Department of Respiratory Medicine Kasukabe Medical Center Kasukabe Saitama Japan; ^2^ Department of Thoracic Surgery Kasukabe Medical Center Kasukabe Saitama Japan

**Keywords:** bronchoscopy, malignant lymphoma, steroid, tracheal stenosis, wheezing

## Abstract

A 88‐year‐old woman with refractory wheezing and a transient response to corticosteroids was diagnosed with primary tracheal B‐cell lymphoma causing airway stenosis. Emergency radiotherapy markedly improved the stenosis. Tracheal lymphoma should be considered in patients with recurrent wheezing despite asthma treatment, even when corticosteroids provide temporary relief.

## Graphical abstract

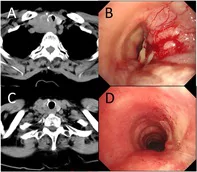

A 88‐year‐old woman presented with refractory wheezing and dyspnea for 2 months despite treatment with inhaled corticosteroids and long‐acting β2‐agonists. She had a history of bronchial asthma and had received short‐term oral prednisolone for asthma exacerbations, with temporary relief. Chest computed tomography (CT) revealed a soft‐tissue mass causing upper tracheal stenosis (Figure 1A). Bronchoscopy revealed a soft, white, haemorrhagic mass 3 cm below the vocal cords (Figure 1B), and forceps biopsies were performed. Emergency radiotherapy was administered to prevent airway obstruction. Bronchoscopic biopsy specimens showed numerous poorly cohesive atypical cells (Figure 2A). The tumour cells were negative for AE1/AE3 (Figure 2B) and diffusely positive for CD20 (Figure 2C) and B‐cell lymphoma 6 (Figure 2D), consistent with B‐cell lymphoma. Positron emission tomography/CT revealed no evidence of lymphoma elsewhere, supporting a primary tracheal origin. Two weeks after the initiation of radiotherapy (30 Gy in 10 fractions), the tracheal stenosis had markedly improved (Figure 1C,D). Systemic chemotherapy is planned. Primary tracheal lymphoma should be considered in patients with recurrent wheezing, even when corticosteroids provide temporary relief [1, 2].

**FIGURE 1:**
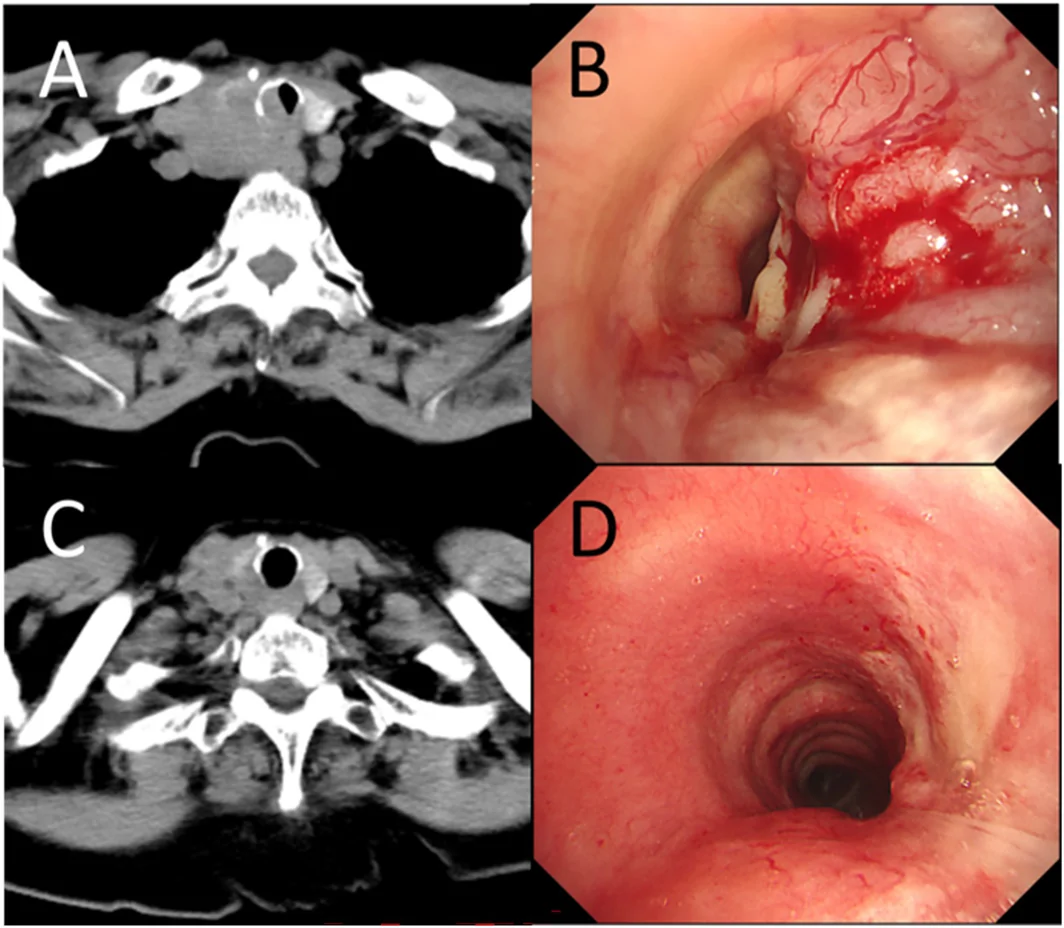
Chest computed tomography (CT) and bronchoscopic findings: (A) Chest CT revealed a soft‐tissue mass causing upper tracheal stenosis. (B) Bronchoscopy revealed a soft, white, haemorrhagic mass 3 cm below the vocal cords. (C, D) Following radiotherapy, the tracheal stenosis improved markedly, as demonstrated by CT (C) and bronchoscopy (D).

**FIGURE 2:**
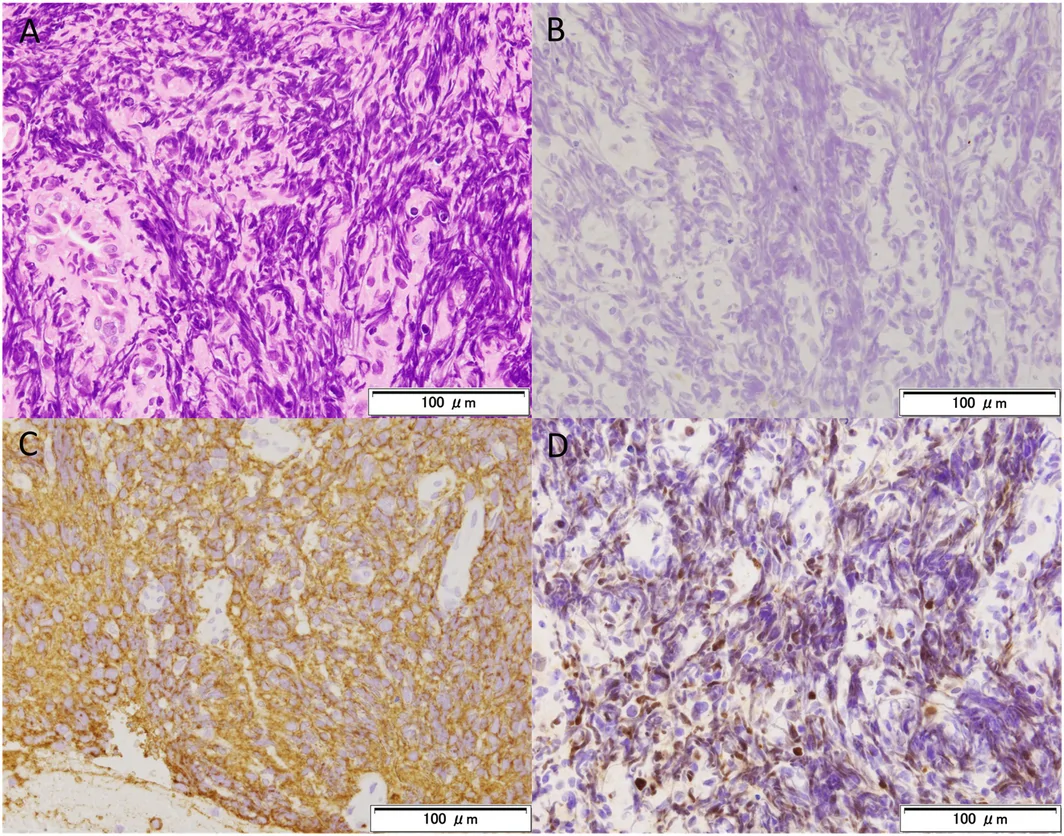
Histopathological and immunohistochemical findings: (A) Bronchoscopic biopsy specimens showed stratified squamous epithelium without atypia and numerous poorly cohesive atypical cells. (B) The tumour cells were negative for AE1/AE3. (C) The tumour cells were diffusely positive for CD20. (D) The tumour cells were diffusely positive for B‐cell lymphoma 6 (BCL6).

## Author Contributions

**Keigo Uchimura:** writing – review and editing, supervision, data curation, writing – original draft. **Sumitaka Kitajima:** data curation, writing – review and editing, writing – original draft. **Makoto Hirai:** writing – review and editing. **Teruaki Ishida:** writing – review and editing.

## Funding

The authors have nothing to report.

## Ethics Statement

The authors have nothing to report.

## Consent

The authors declare that written informed consent was obtained for the publication of this manuscript and accompanying images using the consent form provided by the Journal.

## Conflicts of Interest

The authors declare no conflicts of interest.

## Data Availability

Data sharing not applicable to this article as no datasets were generated or analysed during the current study.
